# The EBV Gastric Cancer Resource (EBV-GCR): A Suite of Tools for Investigating EBV-Associated Human Gastric Carcinogenesis

**DOI:** 10.3390/v15040853

**Published:** 2023-03-27

**Authors:** Mikhail Y. Salnikov, Eric Wang, Erik Christensen, Martin A. Prusinkiewicz, Parisa Shooshtari, Joe S. Mymryk

**Affiliations:** 1 Department of Microbiology and Immunology, Western University, London, ON N6A 3K7, Canada; 2 Department of Pathology and Laboratory Medicine, Western University, London, ON N6A 3K7, Canada; 3 Department of Computer Science, Western University, London, ON N6A 5B7, Canada; 4 Children’s Health Research Institute, Lawson Health Research Institute, London, ON N6A 5W9, Canada; 5 The Ontario Institute for Cancer Research, Toronto, ON M5G 0A3, Canada; 6 Department of Oncology, Western University, London, ON N6A 3K7, Canada; 7 London Regional Cancer Program, Lawson Health Research Institute, London, ON N6A 5W9, Canada; 8 Department of Otolaryngology, Western University, London, ON N6A 5W9, Canada

**Keywords:** Epstein-Barr virus, gastric cancer, TCGA, single-cell RNA sequencing, immune landscape, gene expression, tumor immunology, epigenetic changes, methylation, bioinformatics

## Abstract

Epstein-Barr virus (EBV) causes lifelong infection in over 90% of the world’s population. EBV infection leads to several types of B cell and epithelial cancers due to the viral reprogramming of host-cell growth and gene expression. EBV is associated with 10% of stomach/gastric adenocarcinomas (EBVaGCs), which have distinct molecular, pathological, and immunological characteristics compared to EBV-negative gastric adenocarcinomas (EBVnGCs). Publicly available datasets, such as The Cancer Genome Atlas (TCGA), contain comprehensive transcriptomic, genomic, and epigenomic data for thousands of primary human cancer samples, including EBVaGCs. Additionally, single-cell RNA-sequencing data are becoming available for EBVaGCs. These resources provide a unique opportunity to explore the role of EBV in human carcinogenesis, as well as differences between EBVaGCs and their EBVnGC counterparts. We have constructed a suite of web-based tools called the EBV Gastric Cancer Resource (EBV-GCR), which utilizes TCGA and single-cell RNA-seq data and can be used for research related to EBVaGCs. These web-based tools allow investigators to gain in-depth biological and clinical insights by exploring the effects of EBV on cellular gene expression, associations with patient outcomes, immune landscape features, and differential gene methylation, featuring both whole-tissue and single-cell analyses.

## 1. Introduction

Epstein-Barr virus (EBV) is a gamma-herpesvirus estimated to infect 90% of the world’s population [1]. EBV infects B lymphocytes and mucosal epithelial cells, influencing their cellular differentiation and growth [2,3,4]. EBV uses a variety of immune evasion strategies to establish lifelong latent infections [5]. Furthermore, EBV expresses several oncogenic proteins and microRNAs (miRNAs) [6,7] that are mechanistically associated with multiple types of cancers, including Burkitt’s and other lymphomas, nasopharyngeal carcinomas, and EBV-associated gastric adenocarcinomas (EBVaGCs) [8]. Overall, EBV infections account for 1.5% of all human cancer cases worldwide [9].

It is estimated that EBV is the causative agent of around 10% of all gastric cancer (GC) cases worldwide, though relative proportions differ by region [10,11]. Furthermore, EBVaGCs are molecularly and pathologically distinct entities from EBV-negative GCs (EBVnGCs), with higher survival rates, male-dominant incidence, genome-wide promoter hypermethylation, increased T cell infiltration, as well as higher levels of MHC-I and MHC-II expression [12,13,14,15,16]. Additionally, several EBV-associated proteins and 44 miRNAs originating from the Bam-HI A rightward transcripts (miR-BARTs) are consistently expressed in EBVaGCs, likely contributing to the oncogenesis and progression of EBVaGCs [17,18].

Indeed, a large recent meta-analysis reported that individuals with latent epithelial EBV infections exhibit an 18-fold increased risk of gastric cancer development [19]. Other risk factors include eating salty or spicy foods, frequently drinking coffee and high-temperature drinks, and exposure to wood dust and/or iron filings [20].

As no licensed prophylactic or therapeutic EBV vaccines exist [21], understanding the mechanistic roles that EBV-encoded proteins and miRNAs play in EBVaGC remains a research area of high importance. While a variety of cell line and animal models exist to help understand how EBV manipulates human cells [22,23], many animal models are not robust, and the applicability of these models to humans is unclear [24,25]. These in vitro and in vivo studies benefit from being validated by observations within primary human cancer tissues. However, this often requires a large quantity of methodologically robust clinical data that can provide the statistical power and sample sizes required to validate EBVaGC cancer models.

The Cancer Genome Atlas (TCGA) is a comprehensive, publicly available atlas of genomic, epigenomic, and transcriptomic data from primary, surgically resected human cancers (https://www.cancer.gov/tcga; accessed on 29 August 2022). The outcome of this endeavor is a massive, publicly available, and comprehensive dataset of multidimensional maps of genomic changes in over 11,000 tumor samples from 33 different types of human cancer. Most samples collected to construct the TCGA have complete mRNA and miRNA sequencing and DNA methylation profiling. Additional work has systematically estimated numerous immune landscape features for each TCGA cancer sample [26]. Many TCGA samples include comprehensive clinical data, which allows for the comparison of a variety of clinical variables, such as patient survival [27].

Of the different types of EBV-associated cancers, the TCGA includes stomach adenocarcinoma (STAD) samples from nearly 400 cancer patients [28]. All TCGA STAD samples were surgically resected from treatment-naïve patients to avoid the confounding effects of chemo- and radio-therapeutic treatments on the molecular data. The TCGA STADs can be divided based on their molecular features into EBVaGCs and EBVnGCs, with the latter consisting of four subgroups: microsatellite-instable (MSI) tumors, tumors with chromosomal instability (CIN), genomically stable (GS) tumors, and tumors with DNA polymerase epsilon (POLE) mutations [29]. Along with mRNA and miRNA read counts for cellular genes, both viral mRNA and miRNA read counts are available for EBVaGC samples, as are clinical outcomes, including patient survival, and immune landscape features [26,27]. Although the scope of the data provided by TCGA is vast, several tools are available to help improve its accessibility [30]. However, it is still a daunting task for many researchers to analyze such datasets, especially those without a strong background in bioinformatics. No existing tools allow for detailed comparisons of EBVaGCs with EBV negative GC types.

We recently developed a suite of tools to compare human papillomavirus (HPV)-positive cancers from the TCGA cervical cancer and head and neck cancer cohorts with their HPV-negative counterparts, providing improved access to these important clinically relevant resources [31]. In this manuscript, we introduce a comparable and expanded web-based suite of computational tools, The EBV Gastric Cancer Resource (EBV-GCR), featuring the ability to query and visualize cellular and viral gene expression, immune landscape, survival, and methylation data. The EBV-GCR has been further refined and features a single-cell dataset obtained from Zhang et al. [32], which allows users to visualize differences in single-cell gene expressions among 6 histopathologically unique GC types, as well as among 11 different cell subpopulations. Such an array of analytic and visualization tools is intended as a resource for researchers active in the field of EBV-associated gastric cancers, without any requirements for computational or bioinformatics expertise. The EBV-GCR was developed as a resource to facilitate rapid biological and medical insights via the exploration of the impact of EBV on cellular gene expression, associations with patient survival and immune landscape features, altered gene methylation, and single-cell analyses from primary stomach adenocarcinomas. The web suite of tools can be accessed at https://mymryklab.ca/EBV-GCR/ebvgcr-home/, and the standalone version can be downloaded from the GitHub depository at https://github.com/msaland/EBV-GCR-Suite (deposited 5 March 2023).

## 2. Materials and Methods

### 2.1. Implementation and Software

The web server has been deployed on an Amazon Elastic Compute Cloud (EC2) virtual machine instance running Ubuntu version 22.04, available through Amazon Web Services (AWS). The backend of the server is running Shiny Server version 1.5.20.1002, as well as R version 4.2.2. The server setup was performed in agreement with the methods outlined by Charles Bordet (https://www.charlesbordet.com/en/guide-shiny-aws/, accessed on 25 August 2022). The following packages, along with their respective dependencies, have been installed: corrplot version 0.92, corto version 1.1.11, cowplot version 1.1.1, dplyr version 1.0.10, DT version 0.27, gdata version 2.18.0.1, ggplot2 version 3.4.0, ggpubr version 0.5.0, RColorBrewer version 1.1.3, scales version 1.2.1, Seurat version 4.3.0, shiny version 1.7.4, shinyjqui version 0.4.1, shinyjs version 2.1.0, shinythemes version 1.2.0, survival version 3.5.0, survminer version 0.4.9, and tidyverse version 1.3.2. The service is available 24 h/day, 7 days/week at https://mymryklab.ca/EBV-GCR/ebvgcr-home/, excluding downtime for maintenance and software updates. Tutorial videos for tools are available on the home page, under the section titled “Video Guides”. A standalone version of EBV-GCR for the Windows operating system can be downloaded from https://github.com/msaland/EBV-GCR-Suite.

### 2.2. Sample Collection and Ethics

All data were downloaded from The Cancer Genome Atlas (TCGA) via the Broad Genome Data Analysis Center’s Firehose server (https://gdac.broadinstitute.org/, accessed on 2 March 2017) or other publicly available sources as noted below; as a result, no ethical approval was needed. Table 1 lists the number of samples used for calculations with each EBV-GCR tool, with the exception of the single-cell analysis tool. Table S1 lists the number and characteristics of samples used for calculations in the single-cell analysis tool.

### 2.3. Data Sources for Cellular mRNA and miRNA Expression Levels, Patient Cohort Composition, and Analysis Workflow

Level 3 mRNA and miRNA expression data for the TCGA STAD datasets were obtained from the Broad Genome Data Analysis Center’s Firehose server (https://gdac.broadinstitute.org/, accessed on 2 March 2017), with the data normalized using the RSEM algorithm. The mRNA datasets feature expression patterns for 20,531 unique genes, whereas the miRNA datasets feature 1046 unique genes. The mRNA dataset is comprised of 30 EBVaGC, 353 EBVnGC (223 CIN, 73 MSI, 50 GS, and 7 POLE), and 35 normal (noncancerous) control tissues. The miRNA dataset comprises 29 EBVaGC, 315 EBVnGC (203 CIN, 59 MSI, 46 GS, and 7 POLE), and 41 normal control tissues. Boxplots were generated using the ggplot2 package (version 3.4.0). The maximum and minimum boxplot values were calculated as the 1.5× upper and lower quartile ranges, respectively. The correlations between the cellular gene mRNA or miRNA expressions and the GC subtypes were determined by sorting the datasets into their respective subsets, with subsequent calculations performed with R’s built-in wilcox.test function, with the conf.level parameter set to 0.95. Patient cellular gene mRNA and miRNA expressions with >50% zero or null values were marked as nonsignificant regardless of the calculated *p*-values. q-values were calculated for each comparison group with a false-discovery rate (FDR) of 5%.

### 2.4. Data Sources for Viral mRNA and miRNA Expression Levels, Patient Cohort Composition, and Analysis Workflow

The EBV viral mRNA expression datasets were obtained from Chakravorty et al. [33]. The EBV viral miRNA expression datasets were obtained from Ungerleider et al. [34]. These datasets build upon the TCGA dataset, featuring expression levels of 93 different viral mRNA genes, as well as the expression of 34 miR-BARTs and 3 miR-BHRF genes. The mRNA dataset features 26 patient observations, whereas the miRNA dataset features 32 patient observations. Correlations between the EBV mRNA or miRNA and cellular gene mRNA or miRNA expressions were determined via R’s built-in cor.test function, with the function being run with the linear relationship and Spearman correlation coefficient arguments and the conf.level parameter set to 0.95. Patient cellular gene mRNA or miRNA and EBV mRNA expressions with >50% zero or null values were marked as nonsignificant regardless of the calculated *p*-value. q-values were calculated for each comparison group with an FDR of 5%. Boxplots and scatterplots were generated using ggplot2 (version 3.4.0). The maximum and minimum boxplot values were calculated as the 1.5× upper and lower quartile ranges, respectively.

### 2.5. Data Sources for Immune Landscape Features, Patient Cohort Composition, and Analysis Workflow

Immune landscape features for the TCGA STAD dataset were obtained from Thorsson et al. [26], which included 53 unique features, as listed in Table 2. Samples were then selected for analysis based on the availability of the clinical data for the corresponding TCGA sample. The mRNA dataset features clinical data for 30 EBVaGC and 353 EBVnGC (223 CIN, 73 MSI, 50 GS, and 7 POLE) samples. The miRNA dataset features clinical data for 29 EBVaGC and 353 EBVnGC (201 CIN, 59 MSI, 49 GS, and 7 POLE) samples. The viral mRNA/miRNA dataset only features clinical data for 26 and 29 EBVaGC patient observations for the mRNAs and miRNAs, respectively. Correlations between the immune landscape features and the cellular gene mRNA and miRNA expressions were determined via R’s built-in cor.test function, with the function being run with the linear relationship and Spearman correlation coefficient arguments and the conf.level parameter set to 0.95. Immune landscape features and cellular mRNA and miRNA expressions with >50% zero or null values were marked as nonsignificant regardless of the calculated *p*-values. q-values were calculated for each comparison group, with an FDR of 5%. Boxplots and scatterplots were generated using ggplot2 (version 3.4.0). The maximum and minimum boxplot values were calculated as the 1.5× upper and lower quartile ranges, respectively.

### 2.6. Data Sources for Patient Survival, Patient Cohort Composition, and Analysis Workflow

The TCGA STAD overall survival (OS) datasets were obtained from Liu et al. [27]. Based on the availability of the OS datasets, the corresponding mRNA dataset features 30 EBVaGC and 328 EBVnGC (220 CIN, 72 MSI, 29 GS, and 7 POLE) patient observations. The corresponding miRNA dataset features 29 EBVaGC and 328 EBVnGC (199 CIN, 58 MSI, 49 GS, and 7 POLE) patient observations. Correlations between survival and cellular mRNA and miRNA expressions were determined via the pairwise_survdiff and Surv functions, available via the survminer (version 0.4.9) and survival (version 3.5.0) packages, respectively. Users have the option of selecting the number of comparison groups, upon which the subsets are broken down by the number of selected quantiles based on the mRNA and miRNA expression levels. Patient cellular gene mRNA and miRNA expressions with >50% zero or null values were marked as nonsignificant regardless of the calculated *p*-values. q-values were calculated for each comparison group, with an FDR of 5%. Kaplan–Meier survival plots were generated using the ggsurvplot function available through the survminer package (version 0.4.9).

### 2.7. Data Sources for DNA Methylation Levels, Patient Cohort Composition, and Analysis Workflow

Level 3 Infinium HumanMethylation450 BeadChip array datasets for the TCGA STAD cohort were obtained from the Broad Genome Data Analysis Centers Firehose server (https://gdac.broadinstitute.org/, accessed on 2 March 2017). The datasets feature the methylation beta values for 395,405 different probes, along with chromosome number and genomic coordinates. The dataset features methylation data for 303 STAD samples, with 29 EBVaGC, 313 EBVnGC (202 CIN, 58 MSI, 46 GS, 7 POLE), and 18 non-cancerous, normal control tissues. Correlations between the probe methylation beta values and the genomic loci were determined via R’s built-in wilcox.test function, with the conf.level parameter set to 0.95. Probe methylation beta with >50% zero or null values were marked as nonsignificant regardless of the calculated *p*-values. q-values were calculated for each comparison group, with an FDR of 5%. Boxplots and line plots were generated using the ggplot2 package (version 3.4.0). The maximum and minimum boxplot values were calculated as the 1.5× upper and lower quartile ranges, respectively.

### 2.8. Data Sources for Single-Cell Analysis, Patient Cohort Composition, and Analysis Workflow

The gene expressions of 48,000 cells obtained from newly diagnosed, treatment-naïve patients were obtained from Zhang et al. [32]. The dataset has 1 diffuse GC (DGC), 5 intestinal GC (IGC), 1 of which is EBVaGC, 3 mixed GC (MGC), 1 chronic gastritis (CG), and 2 normal control (NC) histopathological samples, each of which contributes 4000 cells to the overall dataset. The dataset was filtered down to 31,644 cells via the removal of potential doublets and apoptotic cells using a modified method outlined by Zhang et al. [32], with the filtering quality validated against the paper’s results. For each comparison, the log two-fold changes (Log2FC) of the gene expressions and the associated *p*-values were computed with the FindMarkers function, available through Seurat (version 4.3.0), with the logfc.threshold and min.pct parameters set to 0, and the densify parameter set to true. Violin plots were generated with the VlnPlot function, dimensional reduction plots with the DimPlot function, and feature plots with the FeaturePlot function, all of which are available through Seurat (version 4.3.0). Dimensional reduction plots were generated using the t-distributed stochastic neighbor embedding (t-SNE) reduction algorithm. A total of 12 t-SNE reduced datasets are available: the filtered dataset grouped by the patient histopathological type of the cell subpopulation, and subsets of the filtered dataset for the following 11 cell types: T, epithelial, B, plasma, erythroid, fibroblasts, macrophages/DCs, endothelial, endocrine, granulocyte, and parietal cells. With multiple selected genes, the gene signature was calculated by averaging the transcript levels for each cell, and the *p*-values were combined via Fisher integration of the *p*-values using the fisherp function available through the corto package (version 1.1.11).

## 3. Results

The EBV-GCR web suite is a collection of six unique tools, with each created to explore a variety of molecular or clinical characteristics that may be impacted by the EBV status in GC patients. Such characteristics include differential mRNA and miRNA expression, changes in immune landscape features, overall patient survival, and gene loci methylation. By employing the tools present in the EBV-GCR suite, each of these characteristics can be correlated with EBV status, viral or cellular gene expression, genomic loci, GC histopathological subtype, and cell subpopulations. Table 1 lists the number of samples used for the calculations for each EBV-GCR tool, with the exception of the single-cell analysis tool. Table S1 lists the number of samples used for the computations in the single-cell analysis tool.

### 3.1. Differentially Expressed Gene Analysis

**Figure 1:**
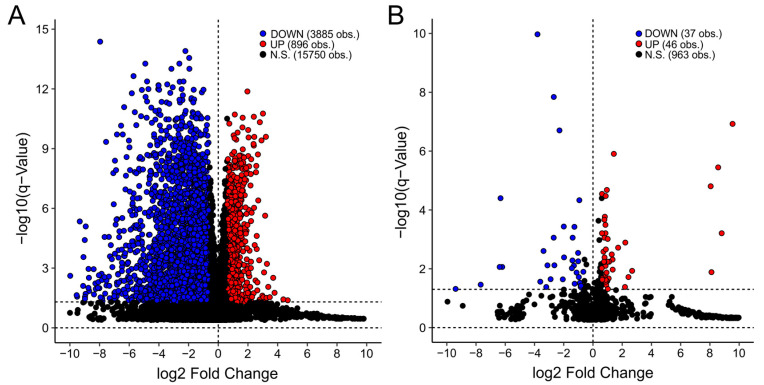
Volcano plots of cellular differentially expressed genes (DEGs) between EBVaGC and EBVnGC samples for the TCGA STAD (**A**) mRNA and (**B**) miRNA datasets. Red dots represent cellular genes that are significantly upregulated in EBVaGCs compared to EBVnGCs. Blue dots represent cellular genes that are significantly down-regulated in EBVaGCs compared to EBVnGCs. Black dots represent cellular genes that are not significantly up- or down-regulated or show less than a 1.5-fold decrease or increase in gene expression in EBVaGCs compared to EBVnGCs. Calculations were performed with an FDR of 5%.

A hallmark of EBV is the ability to reprogram gene expression-infected epithelial and B cells, allowing for its prolonged survival within host cells [35]. In a small subset of infections, this leads to oncogenesis and cancer progression. These cancerous cells continue to express EBV proteins and miRNA [36,37]. As a result, thousands of cellular mRNAs and miRNAs are differentially expressed between EBVaGCs and EBVnGCs (Figure 1), many of which may play roles in immune responses, treatment resistance, cell cycle regulation, etc. The first tool in the EBV-GCR suite is the differential gene expression analysis tool. Users select a gene of interest (GOI), and a table of mRNA expression levels of normalized reads with a corresponding figure illustrating mRNA expression levels and a table of gene expression comparisons among STAD subtypes are generated. This allows users to examine differentially expressed genes (DEGs) among EBVaGC, MSI, GS, CIN, POLE, and normal control tissue. These data can be used to rapidly explore the expressions of STAD-related genes to validate experimental results or promote hypothesis generation.

The DEG tool is available in two versions: one for cellular mRNA genes and one for miRNA gene expression. For either tool, users can select a GOI, upon which two different boxplots are generated, showing the distribution of the expression levels of the GOI for EBVaGC, MSI, CIN, GS, POLE, and normal control samples. Pairwise *p*-values and associated q-values are shown for each comparison, with significant values highlighted. A table containing descriptive statistical information, including the sample size, minimum, maximum, and quartile values, is generated for each of the STAD subtypes. The generated boxplots can be resized via a grey triangle in the corner of the figure, and the figure itself can be downloaded as a raster (PNG) or vector (PDF) graphic. Statistical tables can be downloaded as CSV files, allowing for local data storage and analysis. Comprehensive master lists summarizing the gene expressions of all mRNAs and miRNAs are also downloadable through a link provided on each tool’s webpage.

### 3.2. Correlations between Cellular and Viral Gene Expressions

The vast number of DEGs observed between EBVaGCs and EBVnGCs may be the result of the interaction between EBV-associated viral factors and human genes. Given the ability of EBV to impact gene expression [17,38], we conducted a comprehensive analysis, in which both viral miRNA and mRNA expression data were correlated against cellular mRNA and miRNA expression data. The expressions of numerous cellular mRNAs and miRNAs were significantly correlated, whether positively or negatively, with EBV-associated mRNA (Table 3) and/or miRNA (Table 4) expression. The frequent presence of such correlations may suggest the existence of direct or indirect relationships between EBV and host-cell gene products, which could be further explored mechanistically.

The EBV-GCR suite has four tools for analyzing the correlations between cellular and viral gene expressions. These include the correlations of cellular mRNA with viral mRNA, cellular miRNA with viral mRNA, cellular mRNA with viral miRNA, and cellular miRNA with viral miRNA. For any of these tools, users select a GOI, for which a heatmap is generated, showing both the Spearman correlations and the significance levels of the GOI’s gene expression against 34 miR-BARTs and 3 miR-BHRF genes for the viral miRNA tools and 19 unique viral genes for the viral mRNA versions of the tool. *p*-values and the corresponding q-values are also calculated, with significant values highlighted. The generated heatmap can be resized via a grey triangle in the corner of the figure, and the figure itself can be downloaded as a raster (PNG) or vector (PDF) graphic. Tables summarizing the correlations and significance values can be downloaded as CSV files, allowing for local data storage and analysis. Downloadable lists summarizing the correlations of viral and cellular mRNAs/miRNAs are also available through a link provided on each tool’s webpage.

### 3.3. The Impact of Gene Expression Levels on Overall Survival

**Figure 2:**
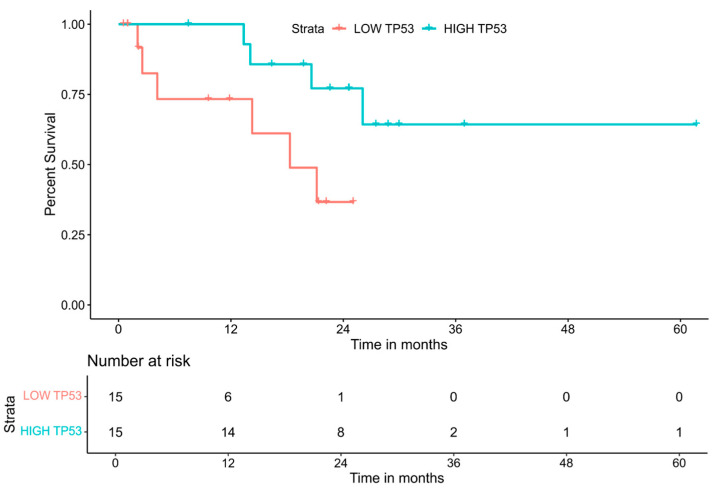
Sample Kaplan–Meier survival curve for p53 (Gene ID: 7157) in the EBVaGC subset of the TCGA STAD cohort. This figure was generated natively by the mRNA version of the overall patient survival tools in the EBV-GCR web suite. The samples are divided into equally sized subsets of high and low expressions of *TP53* transcripts at the median level of expression for *TP53*.

The availability of patient outcome data for a number of TCGA cohorts, including STAD [27], provides an excellent opportunity to explore the impact of altered gene expression on GC patient outcomes. A number of studies have previously employed the TCGA datasets for the elucidation of prognostic genes and gene signatures in a variety of cancers [39,40,41,42]. Our web suite of tools can provide a similar level of analysis; an example of a survival curve generated for p53 (*TP53*) is shown in Figure 2.

There are two versions of the EBV-GCR tool to explore the impact of cellular gene expression on overall patient survival. One explores the impact of cellular mRNA gene expression levels on overall patient survival and the other explores the impact of cellular miRNA gene expression levels on overall patient survival. For either tool, users can select a GOI, as well as the number of comparison groups, with users being limited to 2–4 different comparison groups, and each selection splitting the dataset equally into the number of desired comparison groups. Two comparison groups are titled low and high expression. Three comparison groups are low, mid, and high. Four comparison groups are low, mid-low, high-mid, and high. Upon the selection of both the GOI and the number of comparison groups, a Kaplan–Meier survival curve, along with a risk table, is generated for each of the STAD classifications; EBVaGC, MSI, CIN, GS, and POLE patient groups. Additionally, a table summarizing the pairwise *p*-values and associated q-values is generated, with significant values highlighted. The generated Kaplan–Meier survival curves can be resized via a grey triangle in the corner of the figure, and the figures can be downloaded as a raster (PNG) or vector (PDF) graphic. Tables summarizing the significance values can be downloaded as CSV files, allowing for local data storage and analysis.

### 3.4. The Correlation of Gene Expression Levels with Immune Landscape Features

With the rising clinical emphasis on cancer immunology and immunotherapy in the past decade, it is important to understand the interplay between the immune system in cancer progression and its effects on clinical outcomes [43,44]. The tumor immune landscape of the tumor microenvironment has been shown to play a major role in patient outcomes in GCs [45,46]. In particular, EBVaGCs are “immune hot” tumors with higher levels of MHC I and II expression and cytotoxic T cell infiltration and activation when compared to EBVnGCs. [12,13,15,16,47]. Due to their unique immune features, understanding how EBVaGCs differ in relation to EBVnGCs may help elucidate the underlying mechanisms of EBV-mediated changes in the tumor immune landscape and may eventually result in better treatment options for EBVaGC patients.

**Figure 3:**
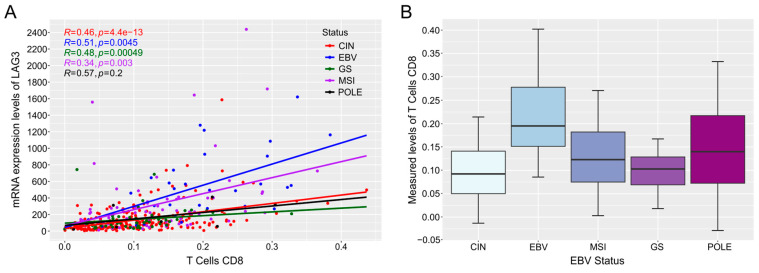
Sample (**A**) correlation plot of LAG3 (Gene ID: 3902) with CD8 T cell fraction (T Cells CD8) in the tumor microenvironment, and (**B**) boxplot representing the distribution of the CD8 T cell fraction across the 5 GC subtypes. This figure was generated natively in the EBV-GCR web suite using the correlation of mRNA gene expression with the immune landscape tool.

There are three versions of the tool to correlate cellular and viral gene expressions with the immune landscape. These are: the correlation of cellular mRNA with immune landscape features, the correlation of cellular miRNA with immune landscape features, and the correlation of viral mRNA and miRNA with immune landscape features. For any of the tools, users select a GOI, which generates a table showing both the Spearman correlation and significance level of the GOI’s gene expression with the 53 immune landscape features (Table 2). The tools for cellular genes generate tables and figures for all samples, whereas the tool featuring viral genes only shows data from the EBVaGC sample group. Significant correlations are highlighted. Users also have the option of selecting an immune landscape feature, which generates a scatterplot depicting correlations between the GOI gene expression levels and the selected immune landscape feature. A boxplot depicting differences in the specific immune landscape feature between the comparison group is also generated, along with a table with pairwise *p*-values. The tool featuring viral genes does not generate either the boxplot or the associated table. Additionally, a table containing descriptive statistics, such as the sample size, minimum, maximum, and quartile values, is generated for each of the subsets exhibited within the correlation plots and boxplots. The generated boxplot and correlation plot can be resized via a grey triangle in the corner of the figure, and the figures can be downloaded as a raster (PNG) or vector (PDF) graphic. Tables summarizing correlations, detailed statistical information, and significance values can be downloaded as CSV files, allowing for local storage and analysis. An example of the sample output of this subset of tools is provided in Figure 3.

### 3.5. Differential Probe Methylation Analysis

**Figure 4:**
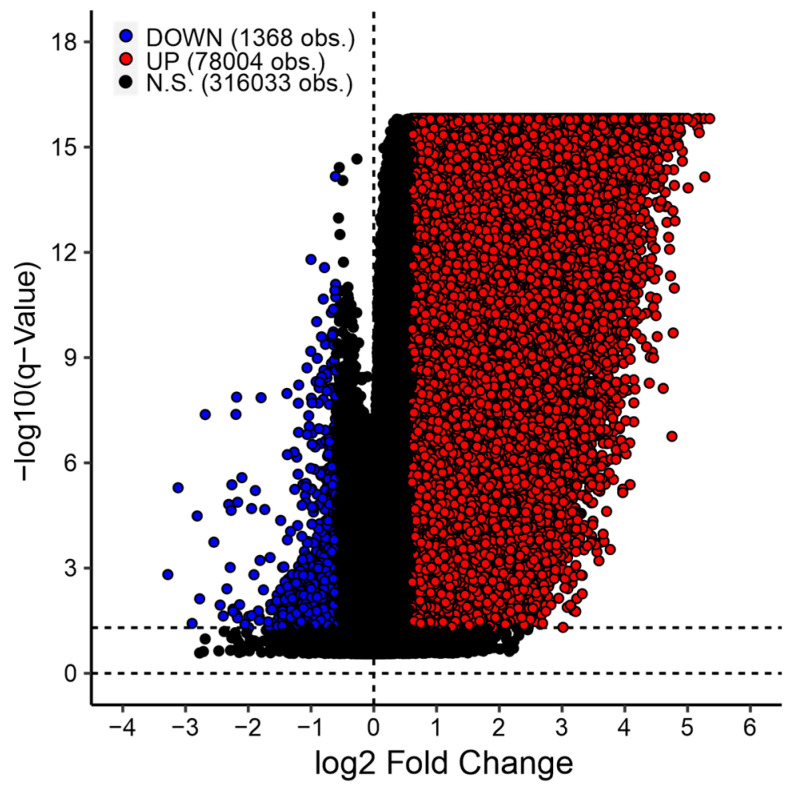
Volcano plot of differentially methylated probes (DMPs) between EBVaGC and EBVnGC samples for the TCGA STAD dataset. Red points represent probes that are significantly up-methylated in EBVaGCs when compared to EBVnGCs. Blue points represent probes that are significantly down-methylated in EBVaGCs when compared to EBVnGCs. Black-colored points represent probes that are not significantly up- or down-methylated or show less than a 1.5-fold decrease or increase in probe methylation in EBVaGC compared to EBVnGC. Calculations were performed with an FDR of 5%.

EBVaGCs are considered to be one of the most hypermethylated tumors, with genome-wide hypermethylation of promoters and genes associated with tumor suppressor functions [29,46] likely contributing to oncogenesis. Indeed, many hypermethylated probes are clearly detected in EBVaGCs from the TCGA STAD cohort, but there is also a fraction of probes that are hypomethylated (Figure 4). The relevance of differential methylation in EBVaGCs has previously been shown [48,49,50], but with our comprehensive, user-friendly tool, studying differential methylation in STAD is possible for a wider range of researchers. Furthermore, when this tool is used in conjunction with the differential gene expression tool, genes that are up- or down-regulated as a result of methylation patterns can be identified.

The tool facilitating differential probe methylation analysis can be used for both the mRNA and miRNA STAD datasets. When users select a GOI, the genomic region and 100,000 base pairs 5′ and 3′ around both sides of the GOI are searched for methylation marks across the encompassed probes. A line plot summarizing the average methylation beta values across EBVaGC, MSI, CIN, GS, POLE, and normal control samples is generated. The line plot displays an arrow representing the coding strand for the GOI, with the left-to-right strand representing the forward orientation and the other strand representing the reverse strand orientation. A comparison table with the *p*-values, q-values, the names of genes associated with the probe, and their chromosomal coordinates is generated, displaying all probes within the selected region. Significant values are highlighted and indicate significant differences in probe methylation. Users then have the option of choosing a probe from the selected region, via an additional dropdown menu, in order to generate a boxplot displaying the methylation beta values across EBVaGC, MSI, CIN, GS, POLE, and normal control samples. The generated line plot and boxplot can be resized via a grey triangle in the corner of the figure, and the figures can be downloaded as a raster (PNG) or vector (PDF) graphic. Tables summarizing the genomic information and significance values can be downloaded as CSV files, allowing for local storage and analysis. A downloadable master list summarizing all differentially methylated probes is also available on each tool’s webpage, which can assist users in identifying DMPs.

### 3.6. Single-Cell Analysis

Bulk RNA sequencing is a technology that allows researchers to look at general expression patterns across tumors but suffers from sampling bias due to intra-tumor heterogeneity, lack of data regarding tumor cellular composition, and lower data fidelity [51,52]. Single-cell RNA sequencing (scRNA-seq) and several other RNA-sequencing techniques have been developed to help remedy some of these issues. In particular, scRNA-seq enables the single-cell resolution of thousands of cells, allowing for the identification of various cell populations, increased resolution of intra-tumoral heterogeneity, and co-expression patterns of genes [53]. The scRNA-seq tool in EBV-GCR is a useful tool to validate results from bulk RNA sequencing and provides a more in-depth understanding of the tumor microenvironment.

**Figure 5:**
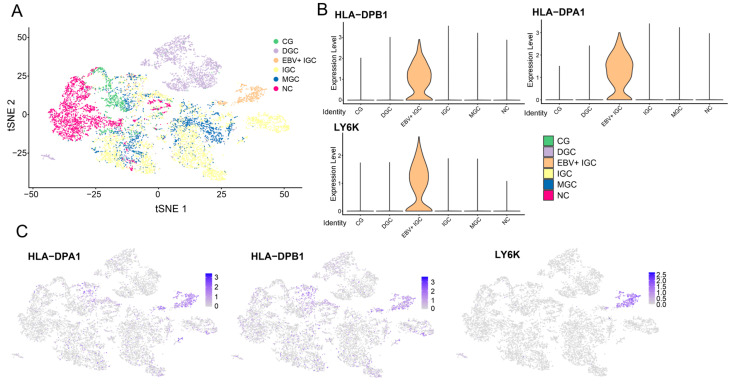
Single-cell analysis data for all epithelial cells grouped by histopathological type. (**A**) Dimensional reduction plot (tSNE) of all epithelial cells, both malignant and healthy. (**B**) Violin plots showing the expression levels of 3 different GOIs (*HLA-DPA1*, *HLA-DPB1*, *LY6K*). (**C**) Feature plot (tSNE) showing relative expression levels of 3 different GOIs (*HLA-DPA1*, *HLA-DPB1*, *LY6K*).

The single-cell analysis tool is based on our analysis of the raw data available from Zhang et al. [32] to compare the relative gene expression in cells from different cell subpopulations found in GCs, as well as across different histopathological types. This tool allows users to select which grouping to use for the single-cell data, as well as one or more GOIs. If only the grouping is selected, a t-SNE dimensional reduction plot is generated, showing the breakdown by patient histopathology or cell type depending on the option selected. If both the grouping and the GOI(s) are selected, in addition to the dimensional reduction plot, a violin plot depicting the relative expressions across the different groupings and a feature plot depicting the relative gene expressions of cells within the confines of the dimensional reduction plot are generated. Additionally, a table summarizing the pairwise *p*-values and associated Bonferroni-adjusted *p*-values is generated, with significant differences in gene expression highlighted. The generated dimensional reduction, violin, and feature plots can be resized via a grey triangle in the corner of the figure, and the figures can be downloaded as a raster (PNG) or vector (PDF) graphic. Tables summarizing the significant values can be downloaded as CSV files, allowing for local storage and analysis. An example of the sample output of this tool is provided in Figure 5.

### 3.7. Example Case Study

**Figure 6:**
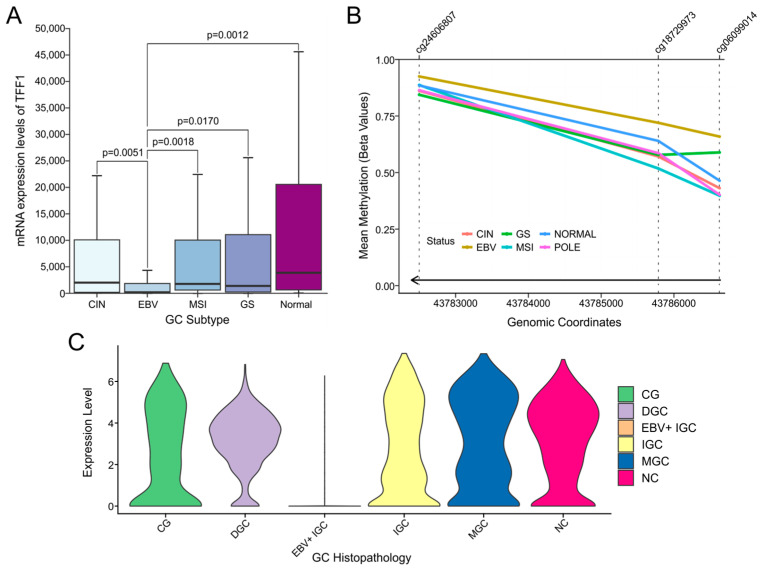
Validation case study for *TFF1*. (**A**) Boxplot of *TFF1* gene expression across EBVaGC and EBVnGC subgroups. Multiple testing was performed with the Conover–Iman test. (**B**) Line plot showing methylation of 3 *TFF1*-associated probes across EBVaGC and EBVnGC subgroups. The line at the bottom of the graph represents gene direction and coverage. (**C**) Violin plot showing relative expression levels of *TFF1* across EBV-positive and EBV-negative histopathological subtypes using the scRNA-seq data.

As an example validation of the utility of the EBV-GCR tool suite, we surveyed the existing literature for a target gene reported in multiple independent studies to be deregulated by EBV in cell culture models and non-TCGA GC datasets. We selected *TFF1*, a trefoil family gene encoding a secretory protein expressed in gastrointestinal mucosa. Multiple reports indicate that *TFF1* expression is downregulated by the EBV infection of AGS or MKN7 gastric cells [54,55,56]. *TFF1* mRNA levels were similarly reduced in non-TCGA cohort-based EBVaGC samples compared to their EBVnGC counterparts [57]. Using the differential gene expression tool for EBV-GCR, a similar, statistically significant downregulation of *TFF1* expression was also observed in the TCGA bulk sequencing data (Figure 6A). Both cell culture infection models and non-TCGA cohort EBVaGC data have identified increased methylation of the *TFF1* gene in the presence of EBV [54,57]. The methylation and silencing can be reversed by 5-aza−2′-deoxycytidine treatment in culture, suggesting that EBV-induced hypermethylation is important for this repression [54]. Using the EBVaGC methylation tool, in good agreement with the published data, an increased level of methylation was observed across the promoter and coding region of *TFF1* in EBVaGCs compared to the other types of GCs, and this was statistically significant in nearly all cases (Figure 6B). Finally, we used the EBV-GCR scRNA-seq analysis tool to demonstrate that *TFF1* expression was significantly reduced in EBV+ IGC epithelial cells vs. EBV-negative gastric epithelial cells (Figure 6C). Thus, the EBV-GCR tools may be helpful for validating experimental results from tissue culture infection models, and they appear to align with the existing clinically derived data.

## 4. Discussion

The vast scale of data provided by the TCGA is an invaluable resource for cancer research, including the study of viral oncogenesis. Although the processed data is freely available via the Broad GDAC Firehose (https://gdac.broadinstitute.org/ accessed on 2 March 2017), the accessibility of such data to researchers without significant computational and bioinformatics skills is limited without substantial investments in time, money, and resources to make these resources easily usable. To help fill this niche, a number of web-based tools have been developed [31,58,59]. The EBV-GCR has been created to provide a wide array of tools for researchers studying EBV oncogenesis in the TCGA STAD cohort. The tools provided by the EBV-GCR include the impact of EBV status on cellular mRNA, miRNA expression levels, and DNA methylation. The EBV-GCR can also perform correlations between cellular gene expression and viral gene expression. Correlation analysis of cellular and viral gene expression with immune landscape features can also be performed. Patient overall survival outcomes can be correlated with cellular gene expression and EBV status. The user-friendly, interactive interface facilitates the conversion of complex data into easy-to-read and intuitive tables and figures, a feature not shared by all analogous web tools.

EBV-GCR also provides the invaluable opportunity of easily downloading both graphical and tabular interpretations for each tool. At the press of a button, users can either download either a vector or raster version of the generated graphic, which users can resize prior to download. The same applies to tabular data, where users can download an easily readable file, which can be used for further analyses and figure generation. Additionally, comprehensive master list files of DEGs, DMPs, and cellular/viral gene expression correlations can also be downloaded for further analysis or reference and allows for the narrowing down of a GOI for further experimental analysis.

To sum up, the EBV-GCR provides an intuitive and data-rich interface for the exploration and interpretation of EBV-dependent changes in gene expression, immune landscape features, patient outcomes, and DNA methylation using molecularly annotated STAD datasets from the TCGA. Single-cell gene expression data can also be examined by the GC histopathological subtype and/or cell subpopulations using datasets from Zhang et al. [32]. Comparisons among EBVaGC, EBVnGC, and normal control samples are possible via a variety of graphs and comprehensive summary tables. Such data can serve as an excellent resource for the validation of experimental observations in model systems and can facilitate the process of novel hypothesis generation. The EBV-GCR is freely accessible at https://mymryklab.ca/EBV-GCR/ebvgcr-home/.

## Figures and Tables

**Table 1:** Number of patient samples analyzed from the TCGA STAD cohort for each EBV-GCR tool, excluding the single-cell analysis tool.

	# of Patient Samples Analyzed											
Patient Subset	mRNA-seq	miRNA-seq	Cellular mRNA vs. Viral mRNA	Cellular miRNA vs. Viral mRNA	Cellular mRNA vs. Viral miRNA	Cellular miRNA vs. Viral miRNA	Immune Comparisons—Cellular mRNA	Immune Comparisons—Cellular miRNA	Immune Comparisons—Viral mRNA/miRNA	Overall Survival—Cellular mRNA	Overall Survival—Cellular miRNA	Methylation
EBV	30	29	26	25	29	30	30	29	26 (mRNA) 29 (miRNA)	30	29	29
MSI	73	59	NA	NA	NA	NA	73	59	NA	72	58	58
CIN	223	203					223	201		220	199	202
GS	60	46					60	46		49	45	46
POLE	7	7					7	7		7	7	7
Normal	35	41					NA	NA		NA	NA	18

^#^ Number.

**Table 2:** List of the 53 immune landscape features available for analysis as part of the EBV-GCR web suite of tools.

Immune Landscape Features *	
Aneuploidy score	Monocytes
B cells memory	Neutrophils
B cells naive	NK cells activated
BCR evenness	NK cells resting
BCR richness	Nonsilent mutation rate
BCR Shannon	No. of segments
CTA score	Plasma cells
Dendritic cells	Proliferation
Dendritic cells activated	Silent mutation rate
Dendritic cells resting	SNV neoantigens
Eosinophils	Stromal fraction
Fraction altered	T cells CD4 memory activated
Homologous recombination defects	T cells CD4 memory resting
IFN gamma response	T cells CD4 Naive
Indel neoantigens	T cells CD8
Intratumor heterogeneity	T cells follicular helper
Leukocyte fraction	T cells gamma delta
Lymphocyte infiltration signature score	T cells regulatory Tregs
Lymphocytes	TCR evenness
Macrophage regulation	TCR richness
Macrophages	TCR Shannon
Macrophages M0	TGF beta response
Macrophages M1	Th1 cells
Macrophages M2	Th17 cells
Mast cells	Th2 cells
Mast cells activated	Wound healing
Mast cells resting	

* BCR, B cell receptor; CTA, cancer testis antigens; IFN, interferon; SNV, single nucleotide variant; TCR, T cell receptor; TGF, transforming growth factor.

**Table 3:** Number of cellular mRNAs and miRNAs positively, negatively, or not significantly correlated with viral EBV mRNAs *.

	Cellular mRNAs			Cellular miRNAs		
Viral mRNA	# of Positive Correlations	# of Negative Correlations	# of Non-Significant Correlations	# of Positive Correlations	# of Negative Correlations	# of Non-Significant Correlations
A73	0	4	20,527	0	0	1046
BALF1	2	0	20,529	0	0	1046
BALF2	2	1	20,528	5	63	978
BALF3	0	0	20,531	0	6	1040
BALF4	1105	1077	18,349	0	7	1039
BALF5	852	952	18,727	2	50	994
BARF0	1	0	20,530	0	0	1046
BARF1	1	0	20,530	0	1	1045
BNRF1	1	0	20,530	0	0	1046
EBER1	1008	1140	18,383	36	4	1006
EBNA-1	4	7	20,520	0	0	1046
LF2	109	114	20,308	0	0	1046
LMP2B	0	0	20,531	0	2	1044
RPMS1	740	526	19,265	5	16	1025

* Note that only viral mRNAs with at least one significant correlation with cellular mRNAs/miRNAs were included. Calculations were performed with an FDR of 5%. ^#^ Number.

**Table 4:** Number of cellular mRNAs and miRNAs positively, negatively, or not significantly correlated with viral EBV miRNAs *.

	Cellular mRNAs			Cellular miRNAs		
Viral mRNA	# of Positive Correlations	# of Negative Correlations	# of Non-Significant Correlations	# of Positive Correlations	# of Negative Correlations	# of Non-Significant Correlations
ebv-miR-BART10-3p	1351	4371	14,809	166	19	861
ebv-miR-BART11-5p	6	19	20,506	5	2	1039
ebv-miR-BART12	0	0	20,531	77	0	969
ebv-miR-BART13-3p	99	239	20,193	20	0	1026
ebv-miR-BART13-5p	328	169	20,034	0	3	1043
ebv-miR-BART14-3p	31	467	20,033	140	0	906
ebv-miR-BART14-5p	391	1864	18,276	237	5	804
ebv-miR-BART15	939	2977	16,615	209	6	831
ebv-miR-BART1-5p	64	988	19,479	13	0	1033
ebv-miR-BART17-3p	519	2971	17,041	124	6	916
ebv-miR-BART17-5p	0	0	20,531	1	0	1045
ebv-miR-BART18-3p	315	212	20,004	0	0	1046
ebv-miR-BART19-3p	0	9	20,522	23	0	1023
ebv-miR-BART19-5p	706	3171	16,654	223	8	815
ebv-miR-BART20-5p	420	1538	18,573	43	0	1003
ebv-miR-BART21-3p	0	0	20,531	117	4	925
ebv-miR-BART22	891	3928	15,712	123	17	906
ebv-miR-BART2-3p	233	1094	19,204	217	6	823
ebv-miR-BART2-5p	506	3218	16,807	155	5	886
ebv-miR-BART3-3p	147	524	19,860	71	0	975
ebv-miR-BART4-3p	116	388	20,027	121	4	921
ebv-miR-BART4-5p	241	1301	18,989	221	9	816
ebv-miR-BART5-5p	815	3885	15,831	162	16	868
ebv-miR-BART6-3p	606	2812	17,113	191	6	849
ebv-miR-BART6-5p	402	1066	19,063	16	0	1030
ebv-miR-BART7-3p	173	696	19,662	121	0	925
ebv-miR-BART7-5p	275	672	19,584	166	2	878
ebv-miR-BART8-3p	858	3550	16,123	129	16	901
ebv-miR-BART8-5p	570	2792	17,169	187	7	852
ebv-miR-BART9-3p	432	1766	18,333	29	6	1011
ebv-miR-BART9-5p	959	3215	16,357	148	4	894

* Note that only viral miRNAs with at least one significant correlation with cellular mRNAs/miRNAs were included. Calculations were performed with an FDR of 5%. ^#^ Number.

## Data Availability

All data are available via the EBV-GCR website or the downloadable standalone Windows version.

## Supplementary Materials

The following supporting information can be downloaded at: https://www.mdpi.com/article/10.3390/v15040853/s1, Table S1: Number of patient samples and cells analyzed for the single-cell analysis tool using the Zhang et al. scRNA-seq cohort [32].
